# 2,4-Dichloro-*N*-*o*-tolyl­benzamide

**DOI:** 10.1107/S1600536809022752

**Published:** 2009-06-20

**Authors:** Aamer Saeed, Rasheed Ahmad Khera, Jim Simpson, Roderick G. Stanley

**Affiliations:** a Department of Chemistry, Quaid-i-Azam University, Islamabad 45320, Pakistan; b Department of Chemistry, University of Otago, PO Box 56, Dunedin, New Zealand

## Abstract

In the title compound, C_14_H_11_Cl_2_NO, the central C—C(O)—N—C amide unit makes dihedral angles of 68.71 (11) and 54.92 (12)°, respectively, with the dichloro­benzene and tolyl rings. The two aromatic rings are inclined at 16.25 (17)°. In the crystal, N—H⋯O hydrogen bonds link mol­ecules into zigzag chains propagating in [001]. C—H⋯Cl contacts link these chains and additional C—H⋯O contacts generate stacks down *b*. Weak C—H⋯π and C—Cl⋯π inter­actions [Cl⋯centroid distance = 3.5422 (15) Å] may also stabilize the structure.

## Related literature

For the biological activity of benzamide derivatives, see: Saeed *et al.* (2008*a* ▶). For related structures, see: Gowda *et al.* (2008 ▶); Saeed *et al.* (2008*b* ▶); Zhou & Zheng (2007 ▶). For reference structural data, see: Allen *et al.* (1987 ▶).
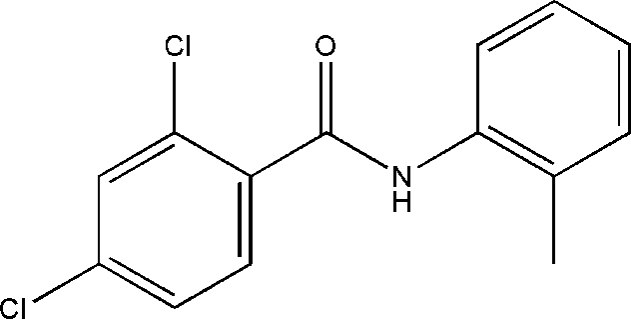


## Experimental

### 

#### Crystal data

C_14_H_11_Cl_2_NO
*M* *_r_* = 280.14
Monoclinic, 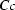

*a* = 22.517 (4) Å
*b* = 6.0405 (9) Å
*c* = 9.6332 (17) Å
β = 104.838 (9)°
*V* = 1266.6 (4) Å^3^
*Z* = 4
Mo *K*α radiation
μ = 0.50 mm^−1^
*T* = 92 K
0.46 × 0.27 × 0.19 mm

#### Data collection

Bruker APEXII CCD diffractometer
Absorption correction: multi-scan (*SADABS*; Bruker, 2006 ▶) *T*_min_ = 0.615, *T*_max_ = 0.91
9889 measured reflections
3475 independent reflections
3195 reflections with *I* > 2σ(*I*)
*R*_int_ = 0.043

#### Refinement

*R*[*F* ^2^ > 2σ(*F* ^2^)] = 0.057
*wR*(*F* ^2^) = 0.158
*S* = 1.15
3475 reflections
164 parameters
2 restraints
H-atom parameters constrained
Δρ_max_ = 1.36 e Å^−3^
Δρ_min_ = −0.62 e Å^−3^
Absolute structure: Flack (1983 ▶), 1248 Friedel pairs
Flack parameter: 0.05 (8)

### 

Data collection: *APEX2* (Bruker, 2006 ▶); cell refinement: *APEX2* and *SAINT* (Bruker, 2006 ▶); data reduction: *SAINT*; program(s) used to solve structure: *SHELXS97* (Sheldrick, 2008 ▶); program(s) used to refine structure: *SHELXL97* (Sheldrick, 2008 ▶) and *TITAN2000* (Hunter & Simpson, 1999 ▶); molecular graphics: *SHELXTL* (Sheldrick, 2008 ▶) and *Mercury* (Macrae *et al.*, 2006 ▶); software used to prepare material for publication: *SHELXL97*, *enCIFer* (Allen *et al.*, 2004 ▶), *PLATON* (Spek, 2009 ▶) and *publCIF* (Westrip, 2009 ▶).

## Supplementary Material

Crystal structure: contains datablocks global, I. DOI: 10.1107/S1600536809022752/tk2478sup1.cif

Structure factors: contains datablocks I. DOI: 10.1107/S1600536809022752/tk2478Isup2.hkl

Additional supplementary materials:  crystallographic information; 3D view; checkCIF report

## Figures and Tables

**Table 1:** Hydrogen-bond geometry (Å, °)

*D*—H⋯*A*	*D*—H	H⋯*A*	*D*⋯*A*	*D*—H⋯*A*
N1—H1⋯O1^i^	0.88	2.04	2.865 (4)	156
C6—H6⋯O1^ii^	0.95	2.55	3.415 (4)	151
C11—H11⋯Cl2^iii^	0.95	2.83	3.680 (3)	150
C14—H14*C*⋯*Cg*2^i^	0.98	2.87	3.528 (4)	125

Symmetry codes: (i) 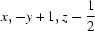; (ii) 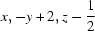; (iii) 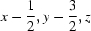. *Cg*2 is the centroid of the C8–C13 ring.

**Fig. 1.**
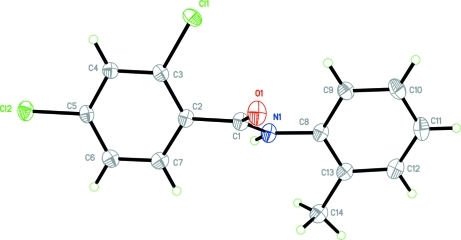
The structure of (I) with displacement ellipsoids for the non-hydrogen atoms drawn at the 50% probability level.

**Fig. 2.**
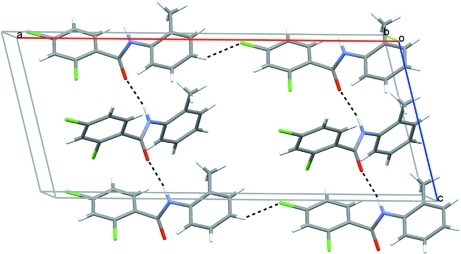
Zig-zag chains of (I) down the c axis. Dashed lines indicate N—H···O hydrogen bonds and C—H···Cl contacts.

**Fig. 3.**
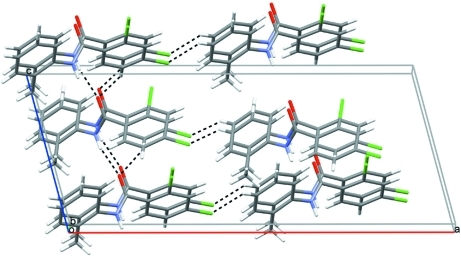
Crystal packing of (I) viewed down the b axis. Dashed lines indicate N—H···O hydrogen bonds as well as C—H···O and C—H···Cl contacts.

## supplementary crystallographic information

### Comment

The biological activity and applications of benzamide derivatives have been described in an earlier paper (Saeed *et al.* 2008*a*). In the title compound, (I) & Fig. 1, the central C2–C1(O1)–N1–C8 amide unit makes dihedral angles of 68.71 (11) ° and 54.92 (12) ° with the C2···C7 and C8···C13 rings, respectively. The two aromatic rings are inclined at 16.25 (17)°. Bond distances within the molecule are normal (Allen *et al.* 1987) and similar to those observed in comparable structures (Gowda *et al.* 2008; Saeed *et al.* 2008*b*; Zhou & Zheng 2007). In the crystal, N1—H1···O1 hydrogen bonds link molecules into zig-zag chains down the *c* axis; Table 1 & Fig. 2. C11—H11···Cl2 contacts link these chains and additional C6—H6···O1 contacts generate three-dimensional stacks down *b*, Fig. 3. C14—H14···π and Cl1···π interactions (Cl···*Cg*1 distance 3.5422 (15) Å; *Cg*1 is the centroid of the C2···C7 ring) may also stabilize the structure.

### Experimental

2,4-Dichlorobenzoyl chloride (5.4 mmol) in CHCl_3_ was treated with 2-methylaniline (21.6 mmol) under a nitrogen atmosphere at reflux for 3 h. Upon cooling, the reaction mixture was diluted with CHCl_3_ and washed consecutively with aq. 1 *M* HCl and saturated aq. NaHCO_3_. The organic layer was dried over anhydrous sodium sulfate and concentrated under reduced pressure. Crystallization of the residue in CHCl_3_ afforded (I) (81%) as colourless crystals: Anal. calcd. for C_14_H_11_Cl_2_NO: C 60.02, H 3.96, N 5.00%; found: C 60.05, H 3.97, N 4.97%.

### Refinement

All H-atoms were positioned geometrically and refined using a riding model with d(C—H) = 0.95 Å, *U*_iso_=1.2*U*_eq_ (C) for aromatic 0.98 Å, *U*_iso_ = 1.5*U*_eq_ (C) for CH_3_ atoms and 0.88 Å, *U*_iso_ = 1.2*U*_eq_ (N) for the NH group. In the final electron density maps, peaks in excess of 1.0 e Å^-3^ were found approximately 0.8 Å from both Cl atoms but no obvious chemical significance could be attached to them; there was no obvious evidence for disorder involving the Cl atoms.

### Crystal data

**Table:**

C_14_H_11_Cl_2_NO	*F*(000) = 576
*M**_r_* = 280.14	*D*_x_ = 1.469 Mg m^−^^3^
Monoclinic, *C**c*	Mo *K*α radiation, λ = 0.71073 Å
Hall symbol: C -2yc	Cell parameters from 5253 reflections
*a* = 22.517 (4) Å	θ = 2.2–32.4°
*b* = 6.0405 (9) Å	µ = 0.50 mm^−^^1^
*c* = 9.6332 (17) Å	*T* = 92 K
β = 104.838 (9)°	Block, colourless
*V* = 1266.6 (4) Å^3^	0.46 × 0.27 × 0.19 mm
*Z* = 4	

### Data collection

**Table:**

Bruker APEXII CCD diffractometer	3475 independent reflections
Radiation source: fine-focus sealed tube	3195 reflections with *I* > 2σ(*I*)
graphite	*R*_int_ = 0.043
ω scans	θ_max_ = 33.1°, θ_min_ = 1.9°
Absorption correction: multi-scan (*SADABS*; Bruker, 2006)	*h* = −34→33
*T*_min_ = 0.615, *T*_max_ = 0.91	*k* = −9→8
9889 measured reflections	*l* = −12→14

### Refinement

**Table:**

Refinement on *F*^2^	Secondary atom site location: difference Fourier map
Least-squares matrix: full	Hydrogen site location: inferred from neighbouring sites
*R*[*F*^2^ > 2σ(*F*^2^)] = 0.057	H-atom parameters constrained
*wR*(*F*^2^) = 0.158	*w* = 1/[σ^2^(*F*_o_^2^) + (0.0952*P*)^2^ + 1.0992*P*] where *P* = (*F*_o_^2^ + 2*F*_c_^2^)/3
*S* = 1.15	(Δ/σ)_max_ < 0.001
3475 reflections	Δρ_max_ = 1.36 e Å^−^^3^
164 parameters	Δρ_min_ = −0.62 e Å^−^^3^
2 restraints	Absolute structure: Flack (1983), 1248 Friedel pairs
Primary atom site location: structure-invariant direct methods	Flack parameter: 0.05 (8)

### Special details

**Table:**

Geometry. All e.s.d.'s (except the e.s.d. in the dihedral angle between two l.s. planes) are estimated using the full covariance matrix. The cell e.s.d.'s are taken into account individually in the estimation of e.s.d.'s in distances, angles and torsion angles; correlations between e.s.d.'s in cell parameters are only used when they are defined by crystal symmetry. An approximate (isotropic) treatment of cell e.s.d.'s is used for estimating e.s.d.'s involving l.s. planes.
Refinement. Refinement of *F*^2^ against ALL reflections. The weighted *R*-factor *wR* and goodness of fit *S* are based on *F*^2^, conventional *R*-factors *R* are based on *F*, with *F* set to zero for negative *F*^2^. The threshold expression of *F*^2^ > σ(*F*^2^) is used only for calculating *R*-factors(gt) *etc*. and is not relevant to the choice of reflections for refinement. *R*-factors based on *F*^2^ are statistically about twice as large as those based on *F*, and *R*- factors based on ALL data will be even larger.

### Fractional atomic coordinates and isotropic or equivalent isotropic displacement parameters (Å^2^)

**Table:**

	*x*	*y*	*z*	*U*_iso_*/*U*_eq_	
O1	0.16543 (13)	0.5847 (4)	0.3277 (3)	0.0250 (5)	
C1	0.17020 (14)	0.5816 (5)	0.2022 (3)	0.0185 (5)	
C2	0.22063 (13)	0.7094 (5)	0.1617 (3)	0.0172 (5)	
C3	0.28213 (14)	0.6506 (5)	0.2168 (3)	0.0173 (5)	
Cl1	0.30103 (4)	0.41955 (12)	0.32639 (7)	0.02356 (18)	
C4	0.32950 (13)	0.7736 (5)	0.1848 (3)	0.0178 (5)	
H4	0.3712	0.7311	0.2212	0.021*	
C5	0.31386 (14)	0.9607 (5)	0.0978 (3)	0.0184 (5)	
Cl2	0.37258 (4)	1.11763 (13)	0.06163 (7)	0.02513 (18)	
C6	0.25310 (15)	1.0223 (5)	0.0405 (3)	0.0211 (5)	
H6	0.2433	1.1492	−0.0192	0.025*	
C7	0.20711 (15)	0.8956 (5)	0.0719 (3)	0.0202 (6)	
H7	0.1654	0.9355	0.0319	0.024*	
N1	0.13248 (12)	0.4699 (5)	0.0939 (3)	0.0197 (5)	
H1	0.1365	0.4894	0.0063	0.024*	
C8	0.08611 (13)	0.3210 (5)	0.1147 (3)	0.0191 (5)	
C9	0.10239 (14)	0.1560 (5)	0.2194 (3)	0.0218 (6)	
H9	0.1435	0.1458	0.2762	0.026*	
C10	0.05858 (17)	0.0073 (6)	0.2407 (4)	0.0272 (7)	
H10	0.0696	−0.1041	0.3123	0.033*	
C11	−0.00132 (17)	0.0223 (6)	0.1568 (4)	0.0291 (7)	
H11	−0.0316	−0.0780	0.1719	0.035*	
C12	−0.01736 (15)	0.1832 (6)	0.0507 (4)	0.0261 (6)	
H12	−0.0584	0.1889	−0.0075	0.031*	
C13	0.02600 (14)	0.3385 (5)	0.0276 (3)	0.0203 (5)	
C14	0.00831 (15)	0.5163 (6)	−0.0857 (4)	0.0261 (6)	
H14A	0.0153	0.6622	−0.0401	0.039*	
H14B	−0.0352	0.5007	−0.1358	0.039*	
H14C	0.0333	0.5012	−0.1548	0.039*	

### Atomic displacement parameters (Å^2^)

**Table:**

	*U*^11^	*U*^22^	*U*^33^	*U*^12^	*U*^13^	*U*^23^
O1	0.0322 (12)	0.0350 (13)	0.0103 (10)	−0.0082 (9)	0.0100 (9)	−0.0035 (8)
C1	0.0219 (12)	0.0242 (13)	0.0107 (12)	−0.0017 (9)	0.0067 (10)	−0.0010 (9)
C2	0.0202 (11)	0.0246 (13)	0.0082 (10)	−0.0028 (9)	0.0060 (9)	−0.0016 (9)
C3	0.0242 (12)	0.0183 (11)	0.0107 (11)	−0.0014 (9)	0.0067 (9)	−0.0010 (9)
Cl1	0.0313 (4)	0.0213 (3)	0.0185 (3)	0.0008 (2)	0.0070 (3)	0.0049 (2)
C4	0.0207 (13)	0.0207 (12)	0.0125 (12)	−0.0008 (9)	0.0048 (10)	0.0008 (9)
C5	0.0230 (12)	0.0197 (11)	0.0139 (13)	−0.0038 (9)	0.0074 (10)	−0.0004 (9)
Cl2	0.0260 (3)	0.0289 (3)	0.0217 (4)	−0.0064 (3)	0.0084 (3)	0.0042 (3)
C6	0.0262 (13)	0.0243 (13)	0.0137 (12)	−0.0005 (10)	0.0068 (11)	0.0018 (10)
C7	0.0225 (13)	0.0270 (14)	0.0115 (13)	0.0012 (10)	0.0053 (10)	0.0014 (10)
N1	0.0225 (11)	0.0289 (12)	0.0092 (10)	−0.0043 (9)	0.0069 (9)	−0.0008 (9)
C8	0.0207 (12)	0.0266 (13)	0.0114 (12)	−0.0019 (10)	0.0067 (10)	−0.0032 (9)
C9	0.0256 (13)	0.0275 (14)	0.0141 (13)	0.0000 (11)	0.0085 (11)	0.0030 (11)
C10	0.0376 (17)	0.0237 (14)	0.0233 (16)	−0.0026 (12)	0.0134 (14)	0.0027 (11)
C11	0.0348 (17)	0.0288 (15)	0.0274 (18)	−0.0101 (13)	0.0149 (14)	−0.0045 (13)
C12	0.0236 (13)	0.0322 (16)	0.0231 (16)	−0.0039 (11)	0.0070 (12)	−0.0050 (13)
C13	0.0224 (13)	0.0261 (13)	0.0139 (13)	0.0008 (10)	0.0073 (10)	−0.0032 (10)
C14	0.0252 (14)	0.0351 (16)	0.0181 (15)	−0.0001 (12)	0.0060 (12)	0.0004 (12)

### Geometric parameters (Å, °)

**Table:**

O1—C1	1.241 (4)	N1—H1	0.8800
C1—N1	1.346 (4)	C8—C9	1.399 (4)
C1—C2	1.505 (4)	C8—C13	1.402 (4)
C2—C3	1.396 (4)	C9—C10	1.388 (5)
C2—C7	1.404 (4)	C9—H9	0.9500
C3—C4	1.397 (4)	C10—C11	1.386 (5)
C3—Cl1	1.736 (3)	C10—H10	0.9500
C4—C5	1.398 (4)	C11—C12	1.389 (5)
C4—H4	0.9500	C11—H11	0.9500
C5—C6	1.388 (4)	C12—C13	1.412 (5)
C5—Cl2	1.733 (3)	C12—H12	0.9500
C6—C7	1.382 (5)	C13—C14	1.510 (5)
C6—H6	0.9500	C14—H14A	0.9800
C7—H7	0.9500	C14—H14B	0.9800
N1—C8	1.431 (4)	C14—H14C	0.9800
			
O1—C1—N1	124.6 (3)	C9—C8—C13	121.3 (3)
O1—C1—C2	120.3 (3)	C9—C8—N1	118.9 (3)
N1—C1—C2	115.1 (3)	C13—C8—N1	119.7 (3)
C3—C2—C7	118.4 (3)	C10—C9—C8	120.2 (3)
C3—C2—C1	120.8 (3)	C10—C9—H9	119.9
C7—C2—C1	120.8 (3)	C8—C9—H9	119.9
C2—C3—C4	121.3 (3)	C11—C10—C9	119.6 (3)
C2—C3—Cl1	120.0 (2)	C11—C10—H10	120.2
C4—C3—Cl1	118.7 (2)	C9—C10—H10	120.2
C3—C4—C5	118.2 (3)	C10—C11—C12	120.3 (3)
C3—C4—H4	120.9	C10—C11—H11	119.8
C5—C4—H4	120.9	C12—C11—H11	119.8
C6—C5—C4	121.8 (3)	C11—C12—C13	121.4 (3)
C6—C5—Cl2	119.9 (2)	C11—C12—H12	119.3
C4—C5—Cl2	118.3 (2)	C13—C12—H12	119.3
C7—C6—C5	118.8 (3)	C8—C13—C12	117.1 (3)
C7—C6—H6	120.6	C8—C13—C14	121.5 (3)
C5—C6—H6	120.6	C12—C13—C14	121.4 (3)
C6—C7—C2	121.5 (3)	C13—C14—H14A	109.5
C6—C7—H7	119.3	C13—C14—H14B	109.5
C2—C7—H7	119.3	H14A—C14—H14B	109.5
C1—N1—C8	123.1 (3)	C13—C14—H14C	109.5
C1—N1—H1	118.5	H14A—C14—H14C	109.5
C8—N1—H1	118.5	H14B—C14—H14C	109.5
			
O1—C1—C2—C3	66.1 (4)	C1—C2—C7—C6	176.2 (3)
N1—C1—C2—C3	−114.1 (3)	O1—C1—N1—C8	−7.1 (5)
O1—C1—C2—C7	−111.4 (4)	C2—C1—N1—C8	173.2 (3)
N1—C1—C2—C7	68.4 (4)	C1—N1—C8—C9	−50.9 (4)
C7—C2—C3—C4	0.3 (4)	C1—N1—C8—C13	130.9 (3)
C1—C2—C3—C4	−177.3 (3)	C13—C8—C9—C10	−1.0 (5)
C7—C2—C3—Cl1	−179.2 (2)	N1—C8—C9—C10	−179.1 (3)
C1—C2—C3—Cl1	3.2 (4)	C8—C9—C10—C11	0.4 (5)
C2—C3—C4—C5	1.2 (4)	C9—C10—C11—C12	0.8 (6)
Cl1—C3—C4—C5	−179.3 (2)	C10—C11—C12—C13	−1.6 (6)
C3—C4—C5—C6	−1.7 (5)	C9—C8—C13—C12	0.3 (5)
C3—C4—C5—Cl2	178.4 (2)	N1—C8—C13—C12	178.4 (3)
C4—C5—C6—C7	0.6 (5)	C9—C8—C13—C14	−180.0 (3)
Cl2—C5—C6—C7	−179.4 (2)	N1—C8—C13—C14	−1.8 (4)
C5—C6—C7—C2	1.0 (5)	C11—C12—C13—C8	1.0 (5)
C3—C2—C7—C6	−1.4 (5)	C11—C12—C13—C14	−178.8 (3)

### Hydrogen-bond geometry (Å, °)

**Table:**

*D*—H···*A*	*D*—H	H···*A*	*D*···*A*	*D*—H···*A*
N1—H1···O1^i^	0.88	2.04	2.865 (4)	156
C6—H6···O1^ii^	0.95	2.55	3.415 (4)	151
C11—H11···Cl2^iii^	0.95	2.83	3.680 (3)	150
C14—H14C···Cg2^i^	0.98	2.87	3.528 (4)	125

Symmetry codes: (i) *x*, −*y*+1, *z*−1/2; (ii) *x*, −*y*+2, *z*−1/2; (iii) *x*−1/2, *y*−3/2, *z*.
